# Evaluation of chimeric recombinant antigens for the serodiagnosis of *Trypanosoma cruzi* in dogs: a promising tool for Chagas disease surveillance

**DOI:** 10.1186/s13071-024-06376-5

**Published:** 2024-07-15

**Authors:** Natália Dantas Fontes, Fernanda Lopes Habib, Leonardo Maia Leony, Natália Erdens Maron Freitas, Ângelo Antônio Oliveira Silva, Filipe Dantas-Torres, Kamila Gaudêncio da Silva Sales, Antônia Cláudia Jácome da Câmara, Vicente Toscano de Araújo-Neto, Leila Denise Alves Ferreira Amorim, Paola Alejandra Fiorani Celedon, Nilson Ivo Tonin Zanchin, Fred Luciano Neves Santos

**Affiliations:** 1 Advanced Public Health Laboratory, Gonçalo Moniz Institute, Oswaldo Cruz Foundation, Salvador, Brazil; 2 Interdisciplinary Research Group in Biotechnology and Epidemiology of Infectious Diseases (GRUPIBE), Gonçalo Moniz Institute, Oswaldo Cruz Foundation (FIOCRUZ-BA), Salvador, Brazil; 3 Laboratory of Immunoparasitology, Department of Immunology, Aggeu Magalhães Institute, Oswaldo Cruz Foundation, Recife, Brazil; 4 Department of Clinical and Toxicological Analysis, Health Sciences Center, Federal University of Rio Grande do Norte, Natal, Brazil; 5 Postgraduate Program in Pharmaceutical Sciences, Federal University of Rio Grande do Norte, Natal, Brazil; 6 Department of Statistics, Institute of Mathematics and Statistics, Federal University of Bahia, Salvador, Brazil; 7 Molecular Biology of Trypanosomatids Laboratory, Carlos Chagas Institute, Oswaldo Cruz Foundation, Curitiba, Brazil; 8 Laboratory of Structural Biology & Protein Engineering, Carlos Chagas Institute, Oswaldo Cruz Foundation, Curitiba, Brazil; 9 Integrated Translational Program in Chagas disease from Fiocruz – Fio-Chagas, Rio de Janeiro, Brazil

**Keywords:** Chagas disease, *Trypanosoma cruzi*, Canine serodiagnosis, Recombinant chimeric antigens, Latent class analysis, Diagnostic performance

## Abstract

**Background:**

Chagas disease (CD), a neglected parasitic disease caused by *Trypanosoma cruzi*, poses a significant health threat in Latin America and has emerged globally because of human migration. *Trypanosoma cruzi* infects humans and over 100 other mammalian species, including dogs, which are important sentinels for assessing the risk of human infection. Nonetheless, the serodiagnosis of *T. cruzi* in dogs is still impaired by the absence of commercial tests. In this study, we investigated the diagnostic accuracy of four chimeric recombinant *T. cruzi* IBMP antigens (IBMP-8.1, IBMP-8.2, IBMP-8.3, and IBMP-8.4) for detecting anti-*T. cruzi* antibodies in dogs, using latent class analysis (LCA).

**Methods:**

We examined 663 canine serum samples, employing indirect ELISA with the chimeric antigens. LCA was utilized to establish a latent variable as a gold standard for *T. cruzi* infection, revealing distinct response patterns for each antigen.

**Results:**

The IBMP (Portuguese acronym for the Molecular Biology Institute of Paraná) antigens achieved area under the ROC curve (AUC) values ranging from 90.9% to 97.3%. The highest sensitivity was attributed to IBMP-8.2 (89.8%), while IBMP-8.1, IBMP-8.3, and IBMP-8.4 achieved 73.5%, 79.6%, and 85.7%, respectively. The highest specificity was observed for IBMP-8.4 (98.6%), followed by IBMP-8.2, IBMP-8.3, and IBMP-8.1 with specificities of 98.3%, 94.4%, and 92.7%, respectively. Predictive values varied according to prevalence, indicating higher effectiveness in endemic settings.

**Conclusions:**

Our findings underscore the remarkable diagnostic performance of IBMP-8.2 and IBMP-8.4 for the serodiagnosis of *Trypanosoma cruzi* in dogs, representing a promising tool for the diagnosis of CD in dogs. These chimeric recombinant antigens may not only enhance CD surveillance strategies but also hold broader implications for public health, contributing to the global fight against this neglected tropical disease.

**Graphical Abstract:**

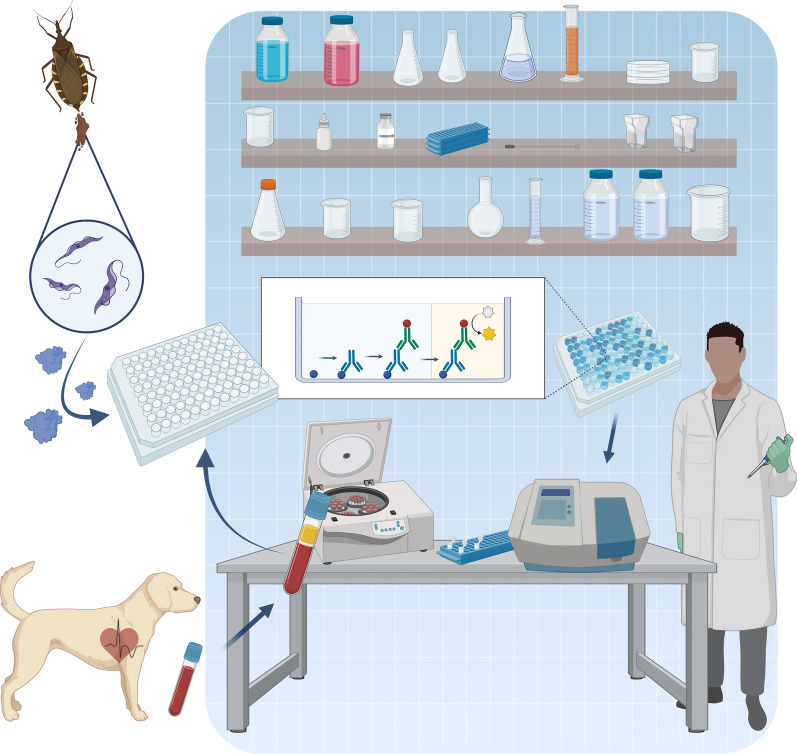

## Background

Chagas disease (CD), caused by the hemoflagellate protozoan *Trypanosoma cruzi*, is a significant neglected parasitic disease affecting humans, especially in Latin American countries. An estimated 5.7–6 million individuals are affected, leading to roughly 10,000 annual fatalities [1]. The primary mode of transmission occurs through the feces of blood-sucking triatomine bugs, placing around 70 million people at risk [1]. Additionally, transmission can happen through blood transfusion, ingestion of contaminated food and beverages, vertical transmission, and accidental laboratory exposure [2]. In recent years, CD has transcended the Americas because of increasing migration, emerging as a global public health concern in various regions worldwide [3, 4].

Clinically, the course of CD can be divided into two phases. The acute phase typically initiates 1–2 weeks post-infection and persists for 2–3 months. It is characterized by high parasitemia and nonspecific clinical signs and symptoms, including pyrexia, tachycardia, and lymphadenopathy [5]. The lifelong chronic phase encompasses two forms: indeterminate and symptomatic. The indeterminate form represents a latent period during which individuals display no clinical signs or symptoms but test positive for the disease through serological testing. Roughly 30–40% of chronically infected individuals progress to the symptomatic form, which can further be categorized as cardiac, digestive, or mixed forms [5]. The diagnosis of CD infection in the chronic phase is complex. Due to intermittent or low parasitemia combined with high anti-*T. cruzi* antibody levels, diagnosis necessitates the use of antigen-antibody detection techniques through laboratory-based methods. Additionally, given the absence of an accurate reference test, the Pan American Health Organization and the World Health Organization conventionally recommend the use of two serological assays based on different antigens and/or methodologies concomitantly to achieve an accurate diagnosis [6].

In addition to humans, *T. cruzi* infects over 100 species of domestic and wild mammals [7]. Domestic animals like chickens, pigs, dogs, and cats heighten the risk of human infection by attracting triatomine bugs [8, 9]. Animals can also acquire infection through blood transfusion, vertical transmission, and orally, via the transmammary route or ingestion of infected insects [10, 11]. Among domestic animals, dogs play a pivotal role in sustaining the domestic and peridomestic cycle of *T. cruzi*. They can be infected through various modes of transmission [12] and may serve as primary host, sentinel, and reservoir of *T. cruzi* in this epidemiological context [10, 13–15]. There is a correlation between seropositivity in humans and dogs in proximity [16]. For instance, adults and children residing in households with seropositive dogs are at a higher risk of infection [16]. Additionally, certain dogs exhibit high parasitemia, attracting vectors and demonstrating high susceptibility to infection [17–19]. Consequently, these animals are recognized as sentinels, as their infection precedes human infection, signaling an active parasite transmission cycle and a potential risk of human infection [20]. Dogs may also develop clinical signs and clinicopathological abnormalities similar to those in humans, rendering them valuable as an experimental model for CD. In severe cases, dogs may experience morphofunctional changes in the cardiac and digestive systems, potentially leading to sudden death [21–24].

Despite the significant role of dogs in the context of CD, there are currently no commercially available tests for detecting the infection in these animals. Previous studies on canine CD have utilized in-house serological assays incorporating native and/or full-length recombinant antigens, resulting in variable diagnostic performance [25]. Recent advances, such as the use of recombinant antigens like the chimeric IBMP proteins (IBMP-8.1, IBMP-8.2, IBMP-8.3, and IBMP-8.4) have shown promising results for the diagnosis of chronic CD in humans [26–35], also exhibiting encouraging performance in dogs [33, 36–38]. A phase I study in dogs demonstrated that all antigens effectively differentiate between positive and negative samples, regardless of whether the infection was experimental or natural. Specifically, IBMP-8.3 and IBMP-8.4 antigens exhibited sensitivity and specificity values exceeding 97.5% [36]. Due to the absence of a reference test for diagnosing CD in humans, we previously adopted a latent class analysis (LCA) approach to mitigate biases arising from the inaccuracies of commercial tests [28]. This approach resulted in high-performance metrics for each IBMP antigen, as validated in several studies [31, 39–41]. The limitations are even greater for dogs because of the lack of commercial tests. Therefore, in this phase II study, we investigated the diagnostic performance of four chimeric recombinant *T. cruzi* IBMP antigens (IBMP-8.1, IBMP-8.2, IBMP-8.3, and IBMP-8.4) for detecting anti-*T. cruzi* antibodies in dogs by in-house ELISA, using latent class analysis (LCA).

## Methods

### Synthesis of chimeric antigens

The chimeric antigens (IBMP-8.1, IBMP-8.2, IBMP-8.3, and IBMP-8.4) employed in this investigation were synthesized following the procedure outlined by Santos et al. [26]. Initially, nucleotide sequences were cloned into the pET28a vector and expressed in *Escherichia coli* BL21-Star DE3. These cells were cultured in lysogeny broth (LB) supplemented with 0.5 M isopropyl-β-d-1-thiogalactopyranoside (IPTG). Subsequently, bacterial cell lysis was achieved using microfluidification, and the antigens were purified through affinity and ion exchange chromatography. The quantification of chimeric antigens was carried out using fluorimetry with the Qubit 2.0 instrument (Invitrogen Technologies, Carlsbad, CA, USA).

### Sample collection

**Fig. 1:**
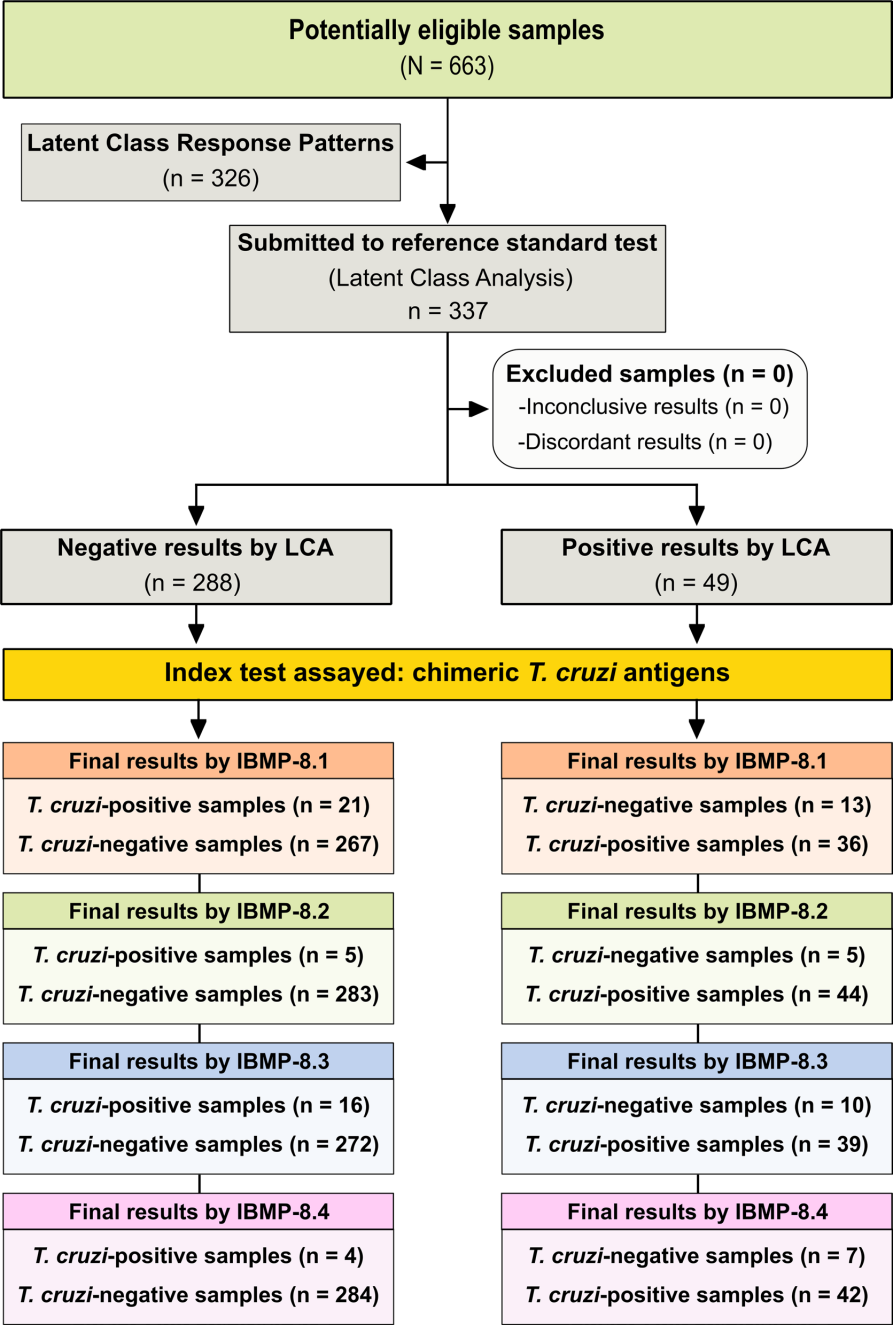
Study analysis design flowchart. This figure illustrates the study design in accordance with the Standards for Reporting of Diagnostic Accuracy Studies (STARD) guidelines. Key abbreviations include N/n (number of samples) and LCA (latent class analysis)

To determine the appropriate sample size, this study employed the statistical program OpenEpi [42], considering a sensitivity of 99%, specificity of 99%, absolute error of 1.5%, and confidence level of 95%. Consequently, a minimum of 169 sera from *T. cruzi*-positive dogs and 169 sera from *T. cruzi*-negative dogs were required. In total, 663 serum samples from dogs were included in the sample panel to assess the diagnostic performance of all four chimeric antigens using ELISA (Fig. 1). These samples were sourced from previous investigations and were collected in various *T. cruzi* endemic settings in Brazil, including Bahia (BA; *n* = 181), Pernambuco (PE; *n* = 402), and Rio Grande do Norte (RN; *n* = 80). All serum samples underwent testing through indirect ELISA, utilizing the four chimeric antigens (IBMP-ELISA). The samples were preassigned unique codes to guarantee a blinded analysis conducted by the operator.

### IBMP-ELISA

Immunoassays were conducted following the procedure outlined by Leony et al. [36]. Flat-bottom 96-well polystyrene microtiter plates (Nunc Maxisorp^®^, USA) were coated with one of the chimeric IBMP antigens at concentrations of 25 ng per well in 100-µl coating buffer (0.05 M carbonate/bicarbonate buffer solution, pH 9.6). Sensitization, stabilization, and blocking were concurrently performed using a synthetic buffer (batch 130,703; WellChampion; Kem-En-Tec Diagnostics A/S, Taastrup, Denmark) according to the manufacturer’s instructions. Serum samples, diluted 1:100 in 0.05 M phosphate-buffered saline (PBS; pH 7.4), were added to the coated wells, and the microtiter plates were incubated at 37 °C for 60 min. Following incubation, the wells were washed five times with 250 µl of wash solution (PBS-Tween; 10 mM sodium phosphate, 150 mM sodium chloride, and 0.5% Tween-20, pH 7.4) to remove non-adsorbed material. Subsequently, the plates were incubated again at 37 °C for 30 min with 100 µl HRP-conjugated anti-dog globulin IgG (Bio-Manguinhos, Fiocruz, Rio de Janeiro-RJ, Brazil) diluted at 1:20,000 (IBMP-8.3) and 1:40,000 (IBMP-8.1, IBMP-8.2, and IBMP-8.4) in PBS. After another wash cycle, 100 µl of chromogenic TBM substrate (Kem-En-Tec Diagnostics A/S, Taastrup, Denmark) was added to each well, and microtiter plates were incubated for 10 min in the dark at room temperature. The colorimetric reactions were interrupted by adding 50 μl of 0.3 M H_2_SO_4_ to each well. Optical density (OD) was measured in a microplate reader equiped with a 450-nm filter (SPECTRAmax 340PC^®^; Molecular Devices, San Jose, CA, USA), with background values subtracted from the measurement experiments.

### Data analysis

LCA was conducted using a statistical model to establish a latent variable and to be subsequently utilized as the gold standard for the diagnosis of *T.cruzi* infection. LCA, a multivariate statistical approach, is based on the analysis of response patterns of categorical indicators (i.e., chimeric antigens) that express a latent categorical construct/variable [43]. Four binary indicators represented the responses (“negative” and “positive”) of the chimeric antigens IBMP-8.1, IBMP-8.2, IBMP-8.3, and IBMP-8.4. LCA model parameters were estimated using maximum likelihood. The set of conditional probabilities estimated by LCA is used to describe the likelihood of a specific response (negative/positive) for a chimeric antigen given the individual’s membership in the corresponding latent class. The interpretation of these estimates are used to label the latent class related to the *T.cruzi* diagnosis. Various criteria were employed to assess the LCA model, including Akaike information criteria (AIC), Bayesian information criteria (BIC), and entropy. Lower AIC and BIC values indicate a better fit, while an entropy value near one indicates high classification quality. Conditional independence was evaluated using bivariate residuals. All analyzes were conducted using Mplus v5.2 software (Muthén & Muthén, Los Angeles, CA, USA). Considering the entire sample panel, 326 out of 663 samples (89 from BA, 195 from PE, and 42 from RN) were randomly selected to define the gold standard for determining the presence of anti-*T. cruzi* antibodies via LCA. The remaining samples were used to estimate diagnostic performance (*n* = 337) for each chimeric antigen based on the previously defined latent class response patterns, along with a corresponding 95% confidence interval. Data were analyzed using GraphPad Prism v 9.5.1 software (GraphPad Software Inc., San Diego, CA, USA). Descriptive data were presented as medians along with interquartile range (IQR) intervals. To assess data normality of ELISA results, the Shapiro-Wilk test was applied. If data did not confirm to the assumption of homogeneity, the Wilcoxon’s signed rank test was utilized. All analyses employed a two-tailed approach, with statistical significance defined as *P* < 0.05. Cutoff values for *T. cruzi* diagnosis were determined by calculating the area under the receiver-operating characteristic (ROC) curve (AUC). AUC values were categorized as low (0.51–0.61), moderate (0.62–0.81), elevated (0.82–0.99), or outstanding (1.0) based on previously established criteria [44]. Results were expressed in index format, denoting the signal-to-cutoff ratio between samples’s OD and the cutoff OD for each microplate. This index is henceforth referred to as reactivity index (RI), with values < 1.00 classified as negative. Samples with RI values within 1.0 ± 10% were categorized as indeterminate, representing a gray zone and considered inconclusive. Various parameters, including sensitivity (Sen), specificity (Spe), accuracy (Acc), and predictive values, were estimated, including their 95% confidence intervals (95% CI). The strength of agreement between LCA and ELISA tests was assessed using Cohen’s *kappa *(κ) analysis, with interpretation as follows: poor (κ ≤ 0), slight (0 < κ ≤ 0.20), fair (0.21 < κ ≤ 0.40), moderate (0.41 < κ ≤ 0.60), substantial (0.61 < κ ≤ 0.80), and near perfect agreement (0.81 < κ ≤ 1.0) [45]. *Trypanosoma cruzi* positive predictive values (PPV) and negative predictive values (NPV) were estimated within a hypothetical prevalence range (from 0.05 to 0.60). In adherence to the Standards for Reporting of Diagnostic Accuracy Studies (STARD) guidelines [46], a flowchart (Fig. 1) and checklist   were prepared to ensure transparency and standardization in reporting methodology and results.

## Results

### Latent class analysis

**Fig. 2:**
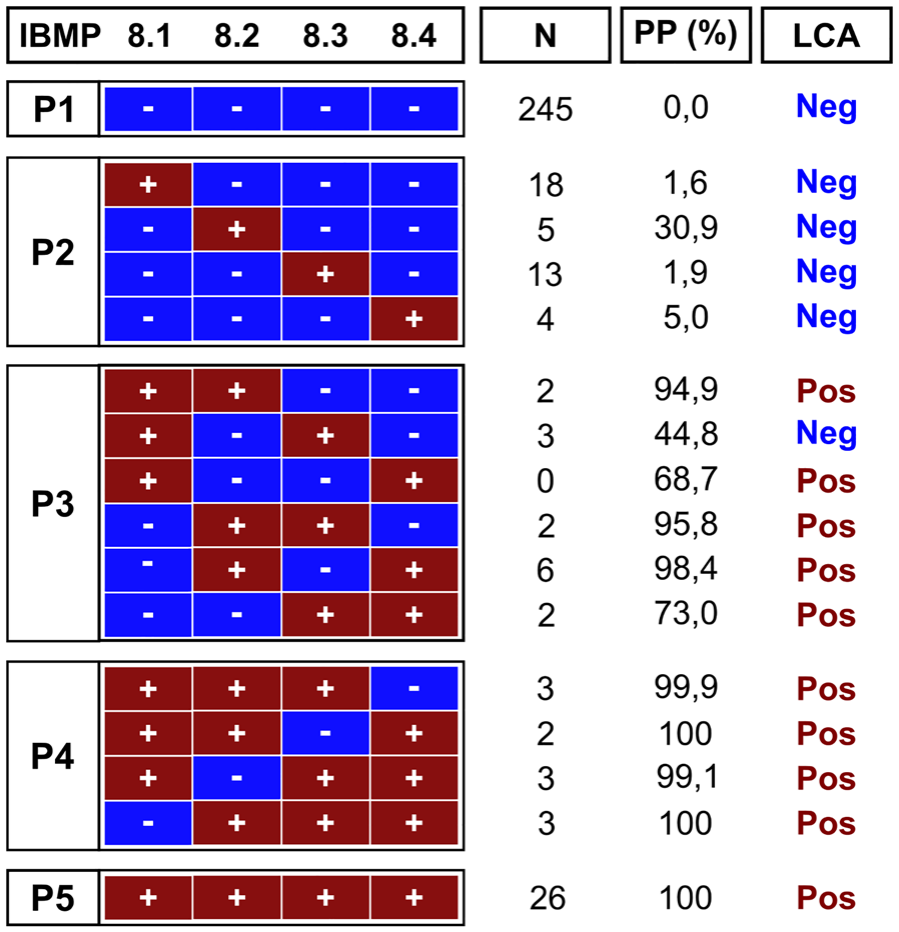
Response patterns for the four chimeric *Trypanosoma cruzi* antigens, a posteriori probabilities for Chagas disease diagnosis from the LCA, and classification of canine serum samples for Chagas disease diagnosis. The samples are denoted as P1–P5 based on their respective response patterns in the chimeric assay. Negative and positive outcomes for a specific chimeric antigen assay are represented by blue and red squares, respectively. Key abbreviations include LCA (latent class analysis), N (number of samples), Neg (negative), Pos (positive), PP (a posteriori probability associated with anti-*T. cruzi* antibody presence)

In this study, a total of 663 samples were included. Approximately 50% of these samples (326 out of 663) were randomly selected to identify and characterize the latent class using LCA based on the response patterns of the four chimeric antigens. A two-class LCA model for CD diagnosis (positive/negative) was selected. The probabilities that each of the chimeric antigens predicted seropositivity in the samples were as follows: 79.1% for IBMP-8.1, 88.1% for IBMP-8.2, 73.4% for IBMP-8.3, and 73.2% for IBMP-8.4. Conversely, the likelihood of misclassifying a negative sample as positive was relatively low, with estimated misclassification rates of 8.3% for IBMP-8.1, 0.6% for IBMP-8.2, 5.1% for IBMP-8.3, and 1.9% for IBMP-8.4. Notably, the overall accuracy of classification was demonstrated by a high entropy value of 0.949. Figure 2 illustrates the response patterns, according to the diagnostic results for positive and negative samples tested using the four chimeric antigens. These patterns were categorized according to the number of negative assays among the four antigens: P1 (no positive results), P2 (25% positive results), P3 (50% positive results), P4 (75% positive results), and P5 (100% positive results). While the number of samples in each pattern varied, the highest frequencies were observed in the P1 (*n* = 245), P2 (*n* = 40), and P5 (*n* = 26) categories. Samples were classified as positive when they tested positive for at least two chimeric antigens (P3-P5), with a posteriori probability (PP) of diagnosis surpassing 68%. However, cases where a sample tested positive for both IBMP-8.1 and IBMP-8.3 were deemed negative because of a PP < 50% (PP = 44.8%). Similarly, samples were classified as negative when they exhibited no positive results or only one positive result for IBMP antigens (P1 and P2), with PP values falling below 31%.

### Individual IBMP-ELISA performance using LCA

LCA classified 288 samples (85.5%) as negative, while 49 samples (14.5%) were predicted to be positive for anti-*T. cruzi* antibodies. Among the samples classified as negative, 245 out of 288 (85.1%) were also negative for all four chimeric antigens by ELISA (Fig. 2). In turn, 40 out of 288 samples (13.9%) exhibited positivity for only one antigen: 18 for IBMP-8.1, five for IBMP-8.2, 13 for IBMP-8.3, and four for IBMP-8.4. The a posteriori probability of these samples being truly positive was < 31%, confirming their correct classification as negative (Fig. 2). The remaining three samples classified as negative were positive for both IBMP-8.1 and IBMP-8.3, with a posteriori probability < 45%. Regarding samples predicted to be positive by LCA, 26 out of 49 (53.1%) were positive for all IBMP antigens, while 12 out of 49 (24.5%) samples exhibited positivity for two antigens: two for IBMP-8.1 + IBMP-8.2, two for IBMP-8.2 + IBMP-8.3, six for IBMP-8.2 + IBMP-8.4, and two for IBMP-8.3 + IBMP-8.4. The probability of these samples being truly positive was > 68.7%, indicating a high likelihood of correct positive classification. The remaining 11 samples classified as positive were positive for three antigens: three for IBMP-8.1 + IBMP-8.2 + IBMP-8.3, two for IBMP-8.1 + IBMP-8.2 + IBMP-8.4, and three for each set of IBMP-8.1 + IBMP-8.3 + IBMP-8.4 and IBMP-8.2 + IBMP-8.3 + IBMP-8.4, with a posteriori probability of these samples being positive exceeding 99% (Fig. 2).

The results of the LCA were used as a gold standard to obtain a reliable estimate of the performance of each chimeric assay. AUC analysis yielded values ranging from 90.9% for IBMP-8.1 and 93.8% for IBMP-8.3 to 97.1% for IBMP-8.2 and 97.3% for IBMP-8.4. These findings indicate a high overall capacity of all four IBMP chimeric antigens to accurately identify *T. cruzi* serological status in dogs.

**Fig. 3:**
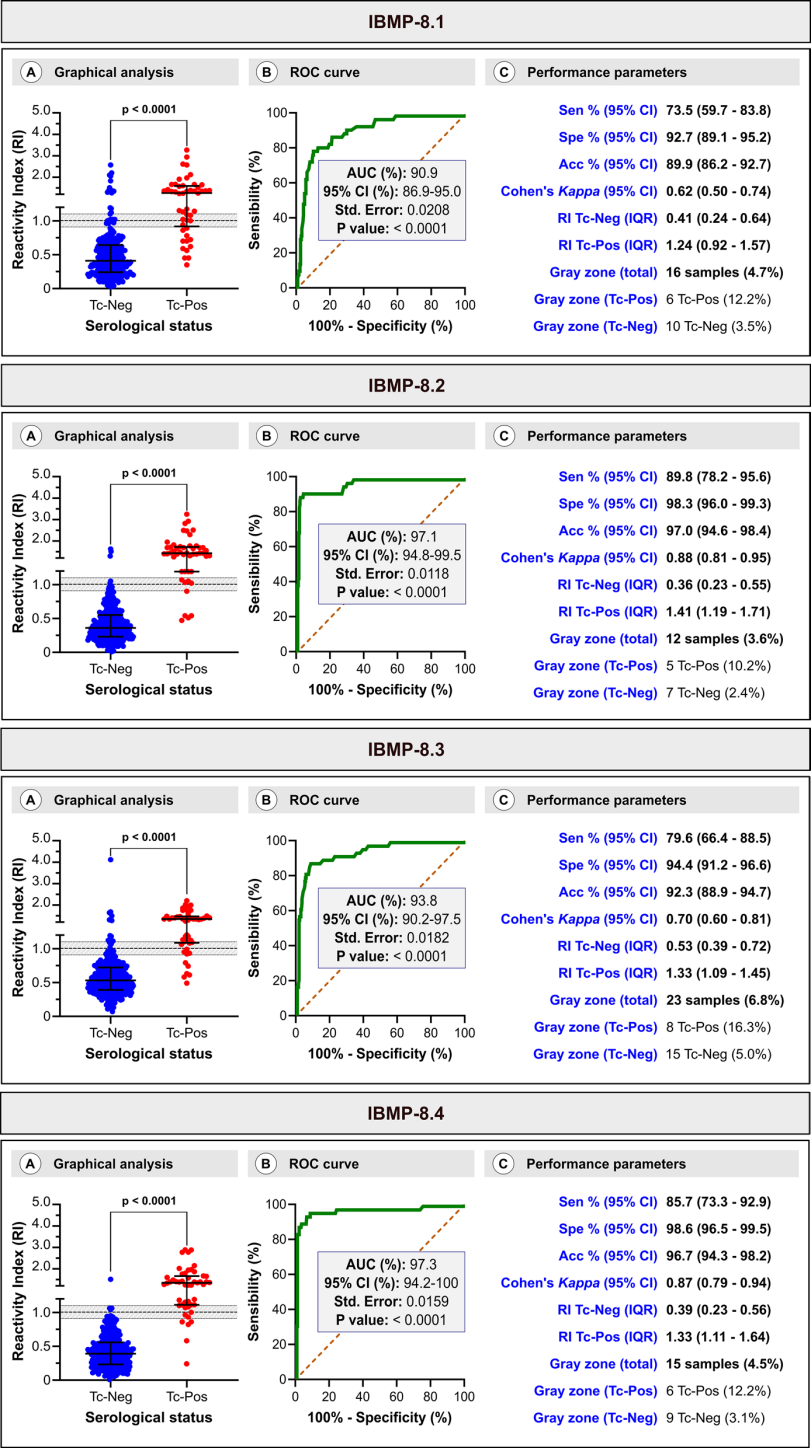
Assessment of chimeric recombinant *Trypanosoma cruzi* antigen performance using ELISA. **A** Graphs illustrating the reactivity index for each antigen assessed against a panel comprising 49 positive and 288 negative samples. The cutoff value is set at 1.0, and the shaded area represents the gray zone. The horizontal lines for each result group denote the medians along interquartile range (IQR). **B** Receiver-operating characteristic curve (ROC) and calculated AUC for each IBMP antigen. **C** Performance parameters for antigens determined using the assays depicted in panel A. Acc: accuracy; AUC: area under the ROC curve; Sen: sensitivity; Spe: specificity; Tc-Neg: *T. cruzi*-negative samples; Tc-Pos: *T. cruzi*-positive samples

Concerning positive sera, those positive for IBMP-8.2 exhibited the highest IgG levels (RI = 1.41; IQR 1.19–1.71), while the lowest levels were observed for IBMP-8.1 (RI = 1.24; IQR 0.92–1.57), with no significant difference between them. Similarly, there were no significant differences in the RIs between IBMP-8.3 (RI = 1.33; IQR 1.09–1.45) and IBMP-8.4 (RI = 1.33; IQR 1.11–1.64). Among the 49 positive samples, IBMP-8.2 demonstrated a sensitivity of 89.8%, with five cases classified as false negative; of these samples, two were also classified as false negatives only for IBMP-8.1. More false-negative results were observed for IBMP-8.1 (13 cases), IBMP-8.3 (10 cases), and IBMP-8.4 (7 cases), with corresponding sensitivity values of 73.5%, 79.6%, and 85.7%, respectively. No statistically significant differences were observed in the sensitivity values. In the negative samples, IBMP-8.2 and IBMP-8.4 showed specificity values ≥ 98%, whereas IBMP-8.1 and IBMP-8.3 produced more false positives, with corresponding specificity values of 92.7% (21 false positives) and 94.4% (16 false positives), respectively. Sera positive for IBMP-8.1 (RI = 0.41), IBMP-8.2 (RI = 0.36), and IBMP-8.4 (RI = 0.39) exhibited the lowest IgG levels without significant differences among them. Conversely, IBMP-8.3-positive sera produced the highest level (RI = 0.53) but with no significant difference compared to other antigens (Fig. 3).

Considering RI values within the range of 1.0 ± 0.10 as the inconclusive gray zone interval for results, our observations revealed that only seven negative samples (2.4%; 7/288) fell into the inconclusive zone when tested with IBMP-8.2. In contrast, 10 samples (3.5%; 10/288) tested with IBMP-8.1, 15 samples (5.2%; 15/288) with IBMP-8.3, and 9 (3.1%; 9/288) with IBMP-8.4 were classified as inconclusive. Among positive samples, we observed the following number of samples in the gray zone: five (10.2%; 5/49) when tested with IBMP-8.2, six (12.2%; 6/49) each when tested with IBMP-8.1 or IBMP-8.4, and eight when tested with IBMP-8.3. Overall analysis showed that 3.6% (12/337) of samples tested with IBMP-8.2, 4.5% (15/337) of samples with IBMP-8.4, 4.7% (16/337) of samples with IBMP-8.1, and 6.8% (23/337) of samples with IBMP-8.3 had RI values within the gray zone.

The assays employing IBMP antigens exhibited an accuracy of 97% for IBMP-8.2, 96.7% for IBMP-8.4, and 94.4% for IBMP-8.3. However, due to the high number of both false-negative and -positive results in samples assayed with IBMP-8.1, the accuracy of this antigen was significantly lower, standing at 89.9%. Cohen's *kappa* index indicated substantial agreement (κ = 0.62 for IBMP-8.1 and κ = 0.70 for IBMP-8.3) and almost perfect agreement (κ = 0.88 for IBMP-8.2 and κ = 0.87 for IBMP-8.4) with the results obtained from LCA. Among the chimeric antigens tested, IBMP-8.4 and IBMP-8.2 demonstrated superior performance, as evidenced from the parameters derived from ROC analysis, accuracy, and Cohen's kappa index. Conversely, IBMP-8.1 displayed the lowest performance. Notably, IBMP-8.2 exhibited the highest sensitivity, while IBMP-8.3 and IBMP-8.4 demonstrated superior specificity.

### Analysis of positive and negative predictive values

**Fig. 4:**
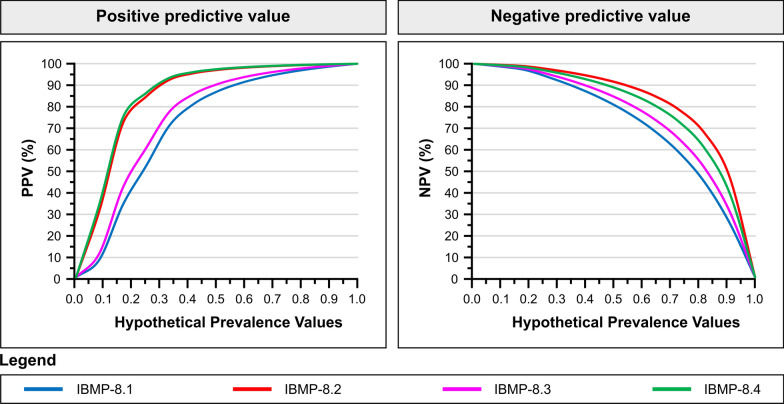
Estimations of positive and negative predictive values across various prevalence scenarios for *Trypanosoma cruzi* seropositivity in dogs, providing insight into the diagnostic accuracy of tests under different epidemiological conditions. NPV: negative predictive value; PPV: positive predictive value

This study also assessed the positive and negative predictive values (PPV and NPV) in the context of *T. cruzi* seropositivity in dogs. To address the varying prevalence of *T. cruzi* seropositivity in dogs across different regions, we applied a range of hypothetical prevalences (1–100%) to simulate a broad spectrum of epidemiological contexts. Figure 4 delineates the relationship between these predictive values and the hypothetical prevalence scenarios. A notable finding was that a decrease in prevalence corresponded with lower PPVs for all chimeric antigens. Nevertheless, the PPV remained comparatively stable for prevalences exceeding 30%, particularly with antigens IBMP-8.2 and IBMP-8.4. These antigens demonstrated an increase in PPV from 95.8% to 99.8% and from 96.3% to 99.8%, respectively, at a 90% prevalence rate. Conversely, an increased prevalence adversely influenced the NPV, especially for IBMP-8.1 and IBMP-8.2.

A thorough analysis of the ratio of false positives/negatives to true positives/negatives across a range of hypothetical prevalence scenarios provided a comprehensive perspective on the performance of testing in different epidemiological settings. As depicted in Table 1, the analysis revealed that the probability of false-negative outcomes, relative to true negatives, was consistently low for all diagnostic tests and across various prevalence scenarios. In contrast, the occurrence of false-positive outcomes, compared to true positives, was significantly more frequent at lower prevalence rates (0.1% and 1%). However, this trend was reversed in areas with higher prevalence levels, where the probability of false positives notably decreased at prevalences of 5% and 10%.

**Table 1:** Probability of false positives relative to true positives and false negatives relative to true negatives across various prevalence levels of *Trypanosoma cruzi* seropositivity in dogs

	IBMP-8.1	IBMP-8.2	IBMP-8.3	IBMP-8.4
Prevalence	False positives: true positives			
0.1%	99.2	18.9	70.3	16.3
1%	9.8	1.87	7.0	1.6
5%	1.9	0.4	1.3	0.3
10%	0.9	0.2	0.6	0.15
Prevalence	False negative: true negatives			
0.1%	< 0.001	< 0.001	< 0.001	< 0.001
1%	0.003	0.001	0.002	0.002
5%	0.015	0.006	0.011	0.008
10%	0.032	0.012	0.024	0.016

## Discussion

Dogs are important sentinels of *T. cruzi* transmission in endemic areas and may exhibit clinical signs and clinicopathological abnormalities similar to those seen in humans. The absence of specific anti-*T. cruzi* antibody detection tests for dogs represents a significant research gap. As a result, studies assessing the seroprevalence of anti-*T. cruzi* antibodies in dogs frequently rely on in-house or human-adapted tests, leading to potential inaccuracies [47–51]. In this study, we evaluated the diagnostic accuracy of four chimeric recombinant *T. cruzi* proteins for identifying anti-*T. cruzi* antibodies in canine serum samples from both endemic and non-endemic regions of Brazil. Our results demonstrated that all IBMP antigens exhibit a high discriminatory ability for identifying positive and negative samples, with AUC values ranging from 90.9 to 97.3%, and the IBMP-8.4 antigen displaying the highest performance. This aligns with our phase I study results, where IBMP-8.4 achieved an AUC value of 100% [36]. Additionally, AUC values for all four proteins exceeded 99% in human sample studies [27, 32–35, 52].

We observed enhanced diagnostic sensitivity with the IBMP-8.2 antigen compared to other antigens, with both IBMP-8.2 and IBMP-8.4 assays demonstrating sensitivities exceeding 80%. These findings contrast those reported in the phase I study, where IBMP-8.3 was more sensitive [36]. In our current analysis, however, both IBMP-8.3 and IBMP-8.1 exhibited the lowest sensitivity values [36]. This divergence in results could be attributed to potential sample misclassification in the phase I study, likely influenced by using in-house or adapted human tests. While two tests had been previously established as a reference standard, the diagnostic performance of the present chimeric antigens could still be influenced by limitations in the precision of these tests. Regarding negative samples, IBMP-8.2 and IBMP-8.4 showed high specificity (≥ 98%), consistent with our previous study [36].

The accuracy values were similar for IBMP-8.2, IBMP-8.3, and IBMP-8.4. Due to the high proportion of misdiagnosed samples detected with IBMP-8.1, its accuracy was lower compared with the other antigens. For human diagnosis, on the other hand, IBMP-8.1 and IBMP-8.4 showed the highest performance [32–35, 52]; therefore, the new commercial lateral flow immunochromatographic assay TR Chagas (Bio-Manguinhos, Fiocruz-RJ, Brazil) uses these two antigens to detect anti-*T. cruzi* antibodies in humans with an accuracy of 91.1% [53]. Although TR Chagas has not yet been validated for diagnosing CD in dogs, its performance was recently evaluated, showing a sensitivity of 94% and a specificity of 91%, according to the ROC curve [54]. The lower performance of IBMP-8.1 for dogs compared to humans might be due to the characteristics of the expressed immunoglobulin VH and VL repertoire in different breeds of dogs compared to those in humans [55]. As such, the use of IBMP-8.1 alone is not recommended for reliable diagnosis in dogs, unless it is used in the latent class model.

This study elucidates the dynamics of predictive values in relation to varying prevalence rates of *T. cruzi* seropositivity in dogs. Notably, there was a decline in PPVs at lower prevalence rates, while stability was observed at higher prevalences. This pattern aligns with the epidemiological characteristics of CD, indicating enhanced accuracy of the chimeric antigens in endemic settings. Conversely, we noted a reduction in NPVs in high-prevalence scenarios. The global escalation of CD, elevating it to a global health issue [4, 56, 57], underscores the urgency of developing an accurate diagnostic test, applicable across all prevalence rates for efficient CD surveillance. Our findings suggest that the IBMP-8.2 and IBMP-8.4 may be promising tools in various epidemiological contexts.

A key limitation of the study was the absence of a validated standard test for preclassifying sera that would be utilized to evaluate the performance of the antigens. To address this limitation, we used a reference array of chimeric *T. cruzi* antigens based on LCA as a surrogate in the absence of a gold standard. LCA provided precise results in the absence of a gold standard. The present study was also limited by testing samples from restricted geographic areas of Brazil, representing a limited number of circulating discrete typing units [58]. Indeed, the reliance on serum samples from dogs in Brazil means these findings might not be universally reproducible in all geographic locations, considering the heterogeneous distribution of different *T. cruzi* strains. It is important that this last limitation be addressed in future studies that consider comprehensive samples from other Brazilian states, especially from the northern region, and from different Latin American endemic countries, where uncommon DTUs can be found. Nevertheless, our analysis confirmed the remarkable performance of these chimeric antigens for detecting anti-*T. cruzi* antibodies in dogs, with IBMP-8.2 and IBMP-8.4 proteins showing higher accuracy. To further improve the performance metrics of the IBMP antigens, our group will conduct additional studies incorporating mixtures of IBMP antigens.

## Conclusions

Our study represents a substantial step towards the development of a reliable serological test for detecting anti-*T. cruzi* antibodies in dogs. Such a test could have applications in both public health (animal health surveillance) and veterinary medicine (CD diagnosis in dogs). In fact, the four chimeric recombinant *T. cruzi* IBMP antigens (especially IBMP-8.2, IBMP-8.3, and IBMP-8.4) displayed remarkable capability in accurately distinguishing positive from negative samples. This finding is particularly significant because of the current lack of specialized commercial tests for canine CD diagnosis. The use of a LCA approach in our analysis further underscores the reliability of these antigens as effective diagnostic tools. The promising results obtained with these chimeric IBMP antigens may improve surveillance and control strategies in areas where CD is endemic, thereby addressing a key challenge of this neglected tropical disease and ultimately to reducing its impact on affected communities.

## Data Availability

Data supporting the conclusions of this study are included in the manuscript, tables and figures.

## Abbreviations

AIC: Akaike information criteria
AUC: Area under the curve
BA: Bahia
BIC: Bayesian information criteria
CD: Chagas disease
CI: Confidence interval
ELISA: Enzyme-linked immunosorbent assay
IBMP: Instituto de Biologia Molecular do Paraná
IPTG: Isopropyl-β-d-1-thiogalactopyranoside
IQR: Interquartile range
LCA: Latent class analysis
OD: Optical density
PBS: Phosphate-buffered saline
PE: Pernambuco
RI: Reactivity index
RN: Rio Grande do Norte
ROC: Receiver-operating characteristic
STARD: Standards for Reporting of Diagnostic Accuracy Studies
TBM: 3,3′,5,5′-Tetramethylbenzidine
